# Induction PD-1 inhibitor toripalimab plus chemotherapy followed by concurrent chemoradiotherapy and consolidation toripalimab for bulky locally advanced non-small-cell lung cancer: protocol for a randomized phase II trial (InTRist study)

**DOI:** 10.3389/fimmu.2023.1341584

**Published:** 2024-01-15

**Authors:** Yu Wang, Lei Deng, Jianyang Wang, Tao Zhang, Wenqing Wang, Xin Wang, Wenyang Liu, Yuqi Wu, Jima Lv, Qinfu Feng, Zongmei Zhou, Jie Wang, Luhua Wang, Zhijie Wang, Nan Bi

**Affiliations:** ^1^ Department of Radiation Oncology, National Cancer Center/National Clinical Research Center for Cancer/Cancer Hospital, Chinese Academy of Medical Sciences and Peking Union Medical College, Beijing, China; ^2^ State Key Laboratory of Molecular Oncology, Department of Medical Oncology, National Cancer Center/National Clinical Research Center for Cancer/Cancer Hospital, Chinese Academy of Medical Sciences and Peking Union Medical College, Beijing, China; ^3^ Department of Radiation Oncology, National Cancer Center/National Clinical Research Center for Cancer/Cancer Hospital & Shenzhen Hospital, Chinese Academy of Medical Sciences and Peking Union Medical College, Shenzhen, China; ^4^ State Key Laboratory of Molecular Oncology, National Cancer Center/National Clinical Research Center for Cancer/Cancer Hospital, Chinese Academy of Medical Sciences and Peking Union Medical College, Beijing, China

**Keywords:** immune checkpoint inhibitor, chemoradiotherapy, induction therapy, programmed cell death-1, non-small-cell lung cancer

## Abstract

**Background:**

Immune checkpoint inhibitors (ICIs) have revolutionized the treatment landscape for locally advanced non-small-cell lung cancer (LA-NSCLC), whereas responses to anti-programmed cell death-1 (PD-1) or anti-programmed death-ligand 1 (PD-L1) are heterogeneous. Though consolidation ICI following concurrent chemoradiotherapy (cCRT) improves survival of NSCLC, this regimen is challenging for patients with bulky tumors due to excessive target volumes and radiation-resistant hypoxia during upfront cCRT, leading to higher risk of pneumonitis and inferior local-regional control. Recent trials have demonstrated neoadjuvant ICI brought greater benefit to stage III than stage I-II NSCLC. Our previous study also supported the therapeutic advantage of 2-cycle induction ICI for patients with bulky unresectable stage III NSCLC. In the context of induction immunotherapy, radiotherapy is more likely to exert immune synergistic effects, reverse anti-PD-1 resistance, and activate abscopal immune responses. Prospective trials to determine the efficacy and safety of induction ICI for bulky LA-NSCLC are necessary.

**Methods:**

This randomized, open-label, two-arm phase II study aims to explore whether 2 cycles of induction anti-PD-1 toripalimab plus chemotherapy can improve progression-free survival (PFS) in bulky LA-NSCLC. Bulky tumors are defined as primary lesion ≥5 cm in greatest dimension or metastatic lymph nodes ≥2 cm in shortest diameter. A total of 50 patients with bulky unresectable stage III NSCLC will be recruited and 1:1 randomized into the experimental arm: 2-cycle induction PD-1 inhibitor toripalimab plus chemotherapy followed by cCRT and consolidation toripalimab; or control arm: 2-cycle induction chemotherapy followed by cCRT and consolidation toripalimab. Patients are stratified by pathology (squamous versus non-squamous). The primary endpoint is PFS. Secondary endpoints are overall survival, overall response rate, disease control rate, duration of response, and incidence of adverse events. Exploratory analyses include PD-L1 expression and liquid biopsy-based biomarker testing, tumor microenvironment profiling at single-cell levels, and quality-of-life assessments.

**Discussion:**

The InTRist study is the first randomized phase II trial to investigate the feasibility of induction anti-PD-1 toripalimab plus chemotherapy followed by cCRT and consolidation toripalimab in bulky LA-NSCLC, providing novel evidence for the synergistic strategy combining anti-PD-1 blockade with radiotherapy to prolong immunotherapy benefits, overcome resistance, and enhance abscopal immune response.

**Clinical trial registration:**

ClinicalTrials.gov, identifier NCT05888402.

## Introduction

Lung cancer remains the leading cause of cancer-related deaths worldwide (1). Non-small-cell lung cancer (NSCLC) represents approximately 80% of all lung cancers, and more than one-third of patients with NSCLC are at locally advanced (LA) stage III (2). Currently, the proportion of LA-NSCLC diagnoses increases annually (3, 4). Definitive concurrent chemoradiotherapy (cCRT) used to be the standard of care (SoC) for unresectable LA-NSCLC (2). Since 2017, the practice-changing results of the PACIFIC trial demonstrated sustained survival benefit from consolidation anti-programmed death ligand-1 (PD-L1) durvalumab in unresectable stage III NSCLC, with the median progression-free survival (PFS) of 16.9 months and median overall survival (OS) of 47.5 months (5, 6). Thereafter, consolidation immune checkpoint inhibitor (ICI) after cCRT became the new SoC for unresectable stage III NSCLC. Notably, stage III NSCLC is a group of diagnoses with heterogeneity, including T1-4 and N0-3, according to the 8th edition of the American Joint Committee on Cancer (AJCC) TNM staging. For patients with larger primary lesions (T3 or T4) or bulky metastatic lymph nodes, cCRT as the initial treatment may bring higher risk of pneumonitis and inferior local-regional control, due to excessive radiation target volumes and radiation-resistant hypoxia inside bulky tumors (7–9). Hence, in real-world settings, 2 cycles of induction chemotherapy for tumor shrinkage, followed by additional 2 cycles of chemotherapy concurrently with thoracic radiotherapy, are common strategy for LA-NSCLC patients with bulky tumors (10, 11).

Furthermore, in the CheckMate 816 trial, neoadjuvant chemoimmunotherapy before surgery brought greater survival benefit to patients with stage IIIA NSCLC than those with stage IB-II diseases, presumably because neoadjuvant ICI combined with chemotherapy could better reduce tumor volumes and contribute to the effectiveness of subsequently radical treatments (12). Thereby, it is reasonable that induction ICI plus chemotherapy before curative-intent cCRT could benefit patients with bulky LA-NSCLC, especially when more patients with stage IIIB/IIIC NSCLC (70%) receiving initial definitive CRT and consolidated ICI in the GEMSTONE-301 trial exhibited unsatisfactory treatment responses and survival outcomes (median PFS of 9.0 months) (13). For patients with bulky unresectable LA-NSCLC, induction chemoimmunotherapy for maximal tumor downsizing could help to decrease target volumes during radiotherapy, reduce the radiation dose of thoracic organs at risk (OARs), and limit the potential risk of radiation-related adverse events (AEs) (9, 10).

More importantly, our prior retrospective study identified that 2 cycles of induction ICI plus chemotherapy could significantly shrink tumor lesions, improve the treatment efficacy, and prolong the survival of patients with bulky LA-NSCLC, with manageable toxic effects (9). After induction chemoimmunotherapy and definitive CRT, median PFS of the patient subset with bulky unresectable LA-NSCLC was 23.8 months, longer than the results from the PACIFIC trial (median PFS was 16.9 months) (5, 9). We also found the disease control rate (DCR; stable disease [SD] + complete response [CR] + partial response [PR]) after 2 cycles of induction chemoimmunotherapy was significantly better than after 4 cycles (97.7% vs 88.4%; P=0.046) and after all cycles (97.7% vs 86.0%; P=0.025), whereas no significant difference in overall response (OR; CR + PR) between different induction treatment cycles (9). Thus, additional cycles of induction systematic therapy beyond the second cycle might result in more progressive disease (PD) (9). In addition, simulated radiation planning based on CT images before and after the first 2 cycles of induction chemoimmunotherapy indicated significant reductions in multiple tumor target volumes and dosimetric parameters (all P<0.001), which were significantly associated with lower risk of all-grade pneumonitis (9). Taken together, our preliminary results suggested the feasibility of 2 cycles of induction ICI plus chemotherapy in bulky LA-NSCLC, and further validation and exploration in prospective clinical trials are warranted.

Toripalimab, a humanized IgG4 anti-programmed cell death-1 (PD-1) monoclonal antibody, combined with chemotherapy as neoadjuvant therapy before radical surgery in stage II/III NSCLC significantly improved event-free survival (EFS) than neoadjuvant chemotherapy (P<0.001), in the randomized, phase III NEOTORCH trial (14). Moreover, in the single-arm, phase II NeoTAP01 study, neoadjuvant toripalimab and chemotherapy followed by surgery in resectable stage III NSCLC also yielded favorable outcomes (1-year and 2-year EFS were 87.8% and 67.9%, respectively) and manageable toxicity (no grade 4 or 5 treatment-related AEs [TRAEs], and the most common grade 3 TRAE was anemia [6.1%]) (15, 16). Given that currently mounting evidence suggests the benefit from neoadjuvant toripalimab in LA-NSCLC (17–19), induction toripalimab before cCRT may also improve the therapeutic effects in bulky unresectable LA-NSCLC.

On the basis of prior findings, we hypothesized that 2 cycles of induction anti-PD-1 toripalimab combined with chemotherapy could benefit patients with bulky LA-NSCLC. Specifically, the tumor shrinkage after induction chemoimmunotherapy was expected to effectively reduce the target volumes and radiation dose on OARs during subsequently cCRT, allowing decreased toxic effects and improved treatment responses. Hence, we planned this first randomized phased II trial to investigate the efficacy and safety of 2 cycles of induction toripalimab with chemotherapy in bulky unresectable LA-NSCLC. After induction treatments, all patients will undergo standard cCRT and consolidation toripalimab for up to 1 year. Meanwhile, we will explore the predictive value of PD-L1 expression, dynamic changes in inflammatory factors including interleukin-6 (IL-6), IL-2, and tumor necrosis factor alpha (TNF-α), and longitudinal liquid biopsy biomarker testing, such as circulating free DNA (cfDNA), circulating tumor DNA (ctDNA), and blood tumor mutational burden (bTMB). Additionally, we will perform single-cell sequencing to profile immunotherapeutic responses across the tumor immune microenvironment. The quality of life (QoL) will be evaluated in all subjects to analyze the long-term impact of treatments. Herein, we report the rationale and study protocol of this randomized phased II trial (the InTRist study), to determine the therapeutic effect of induction and consolidation anti-PD-1 toripalimab in combination with radical radiotherapy for bulky unresectable LA-NSCLC.

## Methods and analysis

### Study design

**Figure 1:**
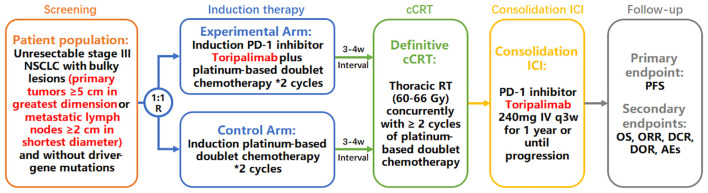
Study design of the InTRist trial.

InTRist is a prospective, randomized, open-label, two-arm (1:1), single-center phased II trial investigating the efficacy and safety of 2 cycles of induction toripalimab plus chemotherapy followed by definitive cCRT and consolidation toripalimab therapy in patients with bulky unresectable LA-NSCLC. The study design is presented in **Figure 1**. Eligible patients are randomized in a 1:1 ratio to the experimental arm (2 cycles of induction toripalimab combined with chemotherapy) or the control arm (2 cycles of induction chemotherapy), stratified according to pathological pattern (squamous cell carcinoma [SCC] versus non-SCC). After the induction systematic therapy, all patients are treated with standard cCRT. Patients who have neither disease progression nor grade ≥ 3 pneumonitis after cCRT will receive consolidation toripalimab for up to 1 year or until clinical disease progression.

### Eligibility criteria

Main inclusion criteria are: 1) Age ≥ 18 years; 2) Eastern Cooperative Oncology Group (ECOG) performance status of 0 or 1 at enrolment; 3) Histologically or cytologically documented NSCLC and present with unresectable stage III disease (LA-NSCLC), per AJCC version 8th; 4) Treatment-naïve patients with bulky tumors, that is, primary lesions ≥ 5 cm in greatest dimension or metastatic lymph nodes ≥ 2 cm in shortest diameter; 5) Whole-body positron emission tomography (PET) scans or contrast-enhanced computed tomography (CT) of the neck, chest, abdomen and pelvis, magnetic resonance imaging (MRI) of the brain, and endobronchial ultrasound with biopsy or fiberoptic bronchoscopy are highly encouraged at diagnosis.

Key exclusion criteria include: 1) Patients harboring sensitizing epidermal growth factor receptor (EGFR) mutations (e.g., exon 19 deletion or exon 21 L858R, exon 21 L861Q, exon 18 G719X, or exon 20 S768I mutation), or anaplastic lymphoma kinase (ALK) mutations; 2) Mixed small cell and NSCLC histology; 3) Previous exposure to anti-PD-1 or PD-L1 antibodies; 4) History of active primary immunodeficiency; 5) Active or prior documented autoimmune diseases or inflammatory disorders; 6) History of allogeneic organ transplantation; 7) Uncontrolled intercurrent illness, including but not limited to, severe infection, congestive heart failure, uncontrolled hypertension, unstable angina pectoris, uncontrolled cardiac arrhythmia, etc., that would limit compliance with study requirement and substantially increase risk of AEs; 8) Unresolved TRAEs of grade 2 or higher; 9) Known allergy or hypersensitivity to any of the study drugs or any of the study drug excipients. Signed written informed consent is needed for all participants.

### Treatment and interventional methods

Eligible patients are 1:1 randomized into the experimental or control arm. In the induction therapy period, patients in the experimental arm are treated with 2 cycles of induction toripalimab combined with chemotherapy (toripalimab 240 mg administered intravenously on day 1 and 22, in combination with platinum-based doublet chemotherapy regimens recommended in the National Comprehensive Cancer Network [NCCN] guidelines) (20). In the control arm, 2 cycles of induction chemotherapy (platinum-based doublet regimens recommended in the NCCN guidelines) are administrated (20). At an interval of 3 to 4 weeks after induction therapy, patients without grade ≥ 3 AEs will further receive cCRT. During the cCRT phase, all patients are treated with identical treatments: definitive cCRT using intensity modulated radiation therapy (IMRT) or volumetric modulated arc therapy (VMAT) techniques with the prescribed dose of 54-66 Gy, concurrently with 2 or more cycles of platinum-containing doublet chemotherapy according to standard guidelines (6, 20). All patients will adopt four-dimensional radiation treatment planning. Gross tumor volume (GTV) of primary tumor (GTVp) is contoured based on post-induction CT scans to take into account changes in tumor size and volume after induction therapy (21). GTV of lymph nodes (GTVn) is defined as any regionally involved nodes on pretreatment PET (standardized uptake value >3) or diagnostic CT (≥1 cm on short axis diameter) scans, using involved field irradiation (20, 22). Clinical target volume (CTV) consists of GTVp plus GTVn with a 0.5 cm margin for microscopic extension. Manual adjustment of CTV is permitted to reduce radiation dose to crucial OARs, such as the spinal cord, when disease is adjacent but not invade the structures. Planning target volume (PTV) comprises CTV with a 0.5 cm margin for set-up errors and organ motion. Furthermore, all patients will undergo contrast-enhanced CT scans at 36-40 Gy (18-20 fractions) during the course of radiation therapy (during-RT). If patients have significant changes in tumor volume or anatomical structure on during-RT CT images, including obvious tumor shrinkage or the regression of atelectasis, the second radiation planning will be adopted after investigators’ evaluation. P2-GTVp, P2-GTVn, P2-CTV, and P2-PTV are delineated on the second CT simulation scan, using the same delineation methods and definitions as the initial radiation planning (23). Patients with neither grade ≥ 3 AEs nor disease progression during and after cCRT will enter the next phase of consolidation ICI treatment: toripalimab 240 mg via intravenous infusion every 3 weeks until confirmed progression or up to 1 year unless there is unacceptable toxicity, withdrawal of consent, or another discontinuation criterion. Lastly, patients will enter the follow-up period (**Figure 1**).

### Objectives and endpoints

The primary objective of this study is to assess the efficacy of induction toripalimab plus chemotherapy compared with induction chemotherapy alone in terms of PFS. Secondary objectives are: 1) to further assess the efficacy of induction toripalimab compared with induction chemotherapy in terms of OS; 2) to evaluate whether the therapeutic effects of induction toripalimab plus chemotherapy, including overall response rate (ORR), DCR, and duration of response (DOR), etc., are superior to induction chemotherapy alone; 3) to investigate the safety and tolerability profile of induction toripalimab plus chemotherapy and compare with induction chemotherapy alone. Exploratory objectives are: 1) to analyze whether induction toripalimab combined with chemotherapy outperforms chemotherapy alone in tumor downsizing; 2) to explore the predictive effects of liquid biopsy biomarkers, such as ctDNA and bTMB, PD-L1 expression levels, tumor immune microenvironment in single-cell analysis, etc.; 3) to describe and evaluate the impact of treatment regimens on health state and QoL using EORTC QLQ-C30 and QLQ-LC13 questionnaires (24, 25). Therefore, the study is planned as a randomized phase II trial with PFS as the primary endpoint. Secondary endpoints are OS, ORR, DCR, DOR, and the incidence of AEs. Exploratory analyses include biomarker studies and QoL assessments.

### Efficacy and toxicity assessment procedures

Efficacy assessments are planned to evaluate at the following landmark timepoints in the both experimental arm and control arm: the 2nd to 4th week after 2 cycles of induction systematic treatment; the 4th week after the completion of cCRT; every 3 months during consolidation ICI therapy for up to 1 year; and thereafter every 3 months during follow-up until disease progression. Evaluations of tumor response to treatment utilize images from PET or contrast-enhanced (preferred) CT of the neck, chest, and abdomen (including the entire liver and both adrenal glands), based on the Response Evaluation Criteria in Solid Tumors (RECIST) version 1.1. In addition to imaging tests, other routine examinations during follow-up include vital signs, performance status, physical examinations, tumor markers and other hematological tests. For patients with disease progression, survival status should be assessed every 2 months after treatment discontinuation. Survival data and the details of first-line and subsequent therapies for cancer will be collected. ORR, DCR, and DOR will be calculated according to the definitions in RECIST v1.1.

In terms of safety assessments, toxic effects will be evaluated at each study visit and AEs will be reported according to the Common Terminology Criteria for Adverse Events (CTCAE) version 5.0. In the induction period, clinical laboratory examinations, including routine blood tests, clinical chemistry, and other hematological tests related to the immune system, will be taken before every cycle of induction therapy. During the cCRT period, routine blood tests and clinical chemistry are performed every week. In the consolidation toripalimab phase, routine blood tests, clinical chemistry, myocardial enzyme spectrum, and electrocardiograms are examined before each administration of toripalimab. Thyroid function tests, urinalysis, coagulation parameters, etc., are also regularly measured during treatment at the times in the assessment schedules and as clinically indicated. Due to the impact of the COVID-19 pandemic (26), the periodic review during the consolidation therapy phase allows for a slight delay, but not exceeds one week. Serious AEs (SAEs) occurring during treatment or until the last dose of consolidation toripalimab, should be recorded and reported immediately and not exceeding 24h after the knowledge of SAEs.

### Exploratory endpoint analyses

**Figure 2:**
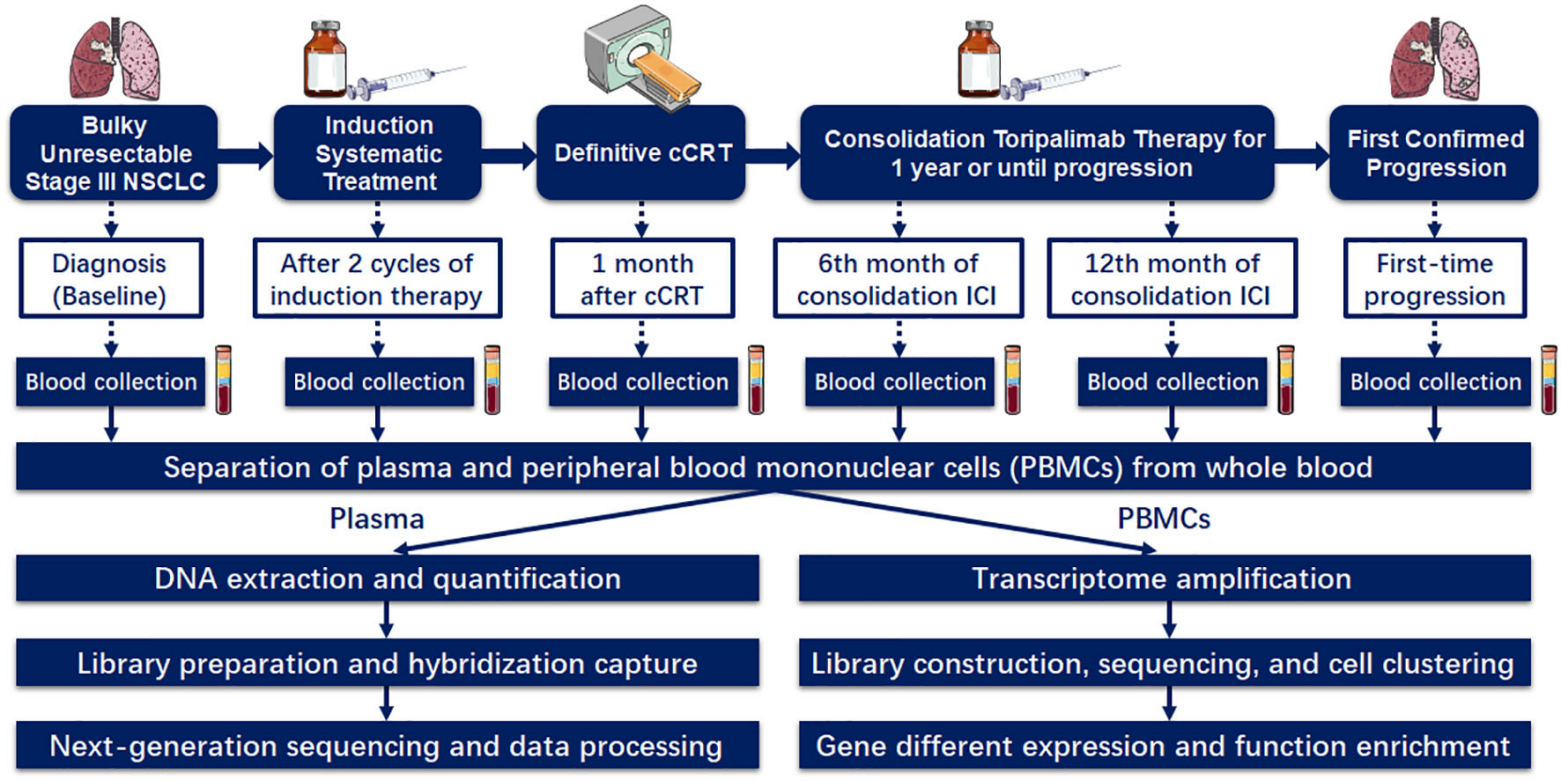
The flow chart of blood sample collection and biomarker assessments.

In the InTRist trial, exploratory analyses include biomarker studies and QoL assessments. Given the rapid development and promising prospects of liquid biopsy technology (27), we will explore whether peripheral biomarkers could predict changes in disease activity as well as survival benefit from ICIs. The flow chart of blood sample collection is shown in **Figure 2**. For all included patients, peripheral blood samples were dynamically collected at the following 6 key timepoints: at diagnosis (baseline), after 2 cycles of induction therapy (before cCRT), 1 month after cCRT completion (before consolidation ICI), the 6th month of consolidation toripalimab, the 12th month of consolidation toripalimab (the end of consolidation ICI), and the first-time confirmed disease progression. After processing whole-blood specimens, plasma is extracted and subjected to next-generation sequencing panel covering 486 cancer-related genes for cfDNA somatic mutations, ctDNA and bTMB assessments. We will further monitor the dynamic changes in inflammatory factors, such as IL-6, IL-2, TNF-α, etc., to profile the inflammatory storm induced by toripalimab, predict the immune response of patients, and early identify potential TRAEs. Hence, plasma obtained at the above landmark timepoints will also be used to assess the concentrations of a panel of relevant cytokines, chemokines, and other immune-related markers. Meanwhile, peripheral blood mononuclear cells (PBMCs) are also isolated from the whole blood for other biomarker assay, such as single-cell sequencing, T cell receptor repertoires, etc., in order to analyze their longitudinal dynamics and explore their clinical predictive value.

Another exploratory endpoint is QoL using EORTC QLQ-C30 and EORTC QLQ-LC13 questionnaires (24, 25). QoL will be evaluated in all patients every 3 months during consolidation ICI therapy and follow-up. Longitudinal QoL data at different timepoints will be compared between the experimental arm (induction toripalimab + chemotherapy) and control arm (induction chemotherapy alone).

### Data analysis and statistical considerations

The primary aim of this trial is to compare PFS between induction chemoimmunotherapy versus induction chemotherapy alone in bulky unresectable LA-NSCLC with sufficient clinical precision. The research hypothesis for this study is that chemoimmunotherapy (toripalimab + chemotherapy) will show improved efficacy compared with chemotherapy alone when given as induction therapy before definitive cCRT to patients with bulky unresectable stage III (primary tumors ≥ 5 cm in greatest dimension or metastatic lymph nodes ≥ 2 cm in shortest diameter). According to our preliminary study investigating induction ICI plus chemotherapy in the LA-NSCLC patient subset with bulky tumors, median PFS was 23.8 months (9). Wu et al. reported that in the phase III GEMSTONE-301 trial, which largely included subjects with bulky LA-NSCLC (stage IIIB/IIIC accounted for 70%), the updated median PFS was 10.5 months for patients receiving induction chemotherapy followed by definitive CRT and consolidation ICI (28). Hence, the two-sided log-rank test with an overall sample size of 46 subjects (23 in the experimental arm and 23 in the control arm) could achieve 80.31% power at a 0.10 significance level (the two-sided alpha error is 0.10, and the beta error is 0.20) to detect a hazard ratio (HR) of 0.441, which is defined as the control group’s median PFS time (10.5 months) divided by the experimental group’s median PFS time (23.8 months), calculated using PASS 15.0.5 software. Based on our prior retrospective analysis, patients with LA-NSCLC at our institution from 2018 to 2022 undergoing induction ICI before radical-intent CRT and consolidation ICI therapy had significantly improved survival than patients without induction ICI, with an HR of 0.30 (95% confidence interval [CI], 0.09-1.00) (29), which provided the supportive evidence for the sample size HR of 0.441 in this phase II trial at our center. This trial will totally last for 50 months of which subject accrual (entry) will occur in the first 12-month period. The accrual pattern across time periods is uniform. Estimating a 10% drop-out rate in the both arms, we set the aim at totally 50 enrolled individuals (25 in the experimental arm and 25 in the control arm).

The intention-to-treat analysis strategy will be employed, and therefore all subjects receiving at least one dose of the assigned treatment will be included into efficacy and safety analyses. PFS is defined as the time from randomization to the date of the first documented disease progression or death without progression. OS is defined as the time from randomization until death from any cause. Survival curves will be calculated using the Kaplan-Meier method and compared by the log-rank test. HRs of PFS and OS will be estimated by the Cox model. Two-sided 95% CI of HRs will be provided.

## Discussion

Consolidation ICI after definitive cCRT has become the new SoC since the PACIFIC trial in 2017, though 52.9% subjects had stage IIIA diseases (6). When more stage IIIB/IIIC NSCLC (70%) patients were enrolled in the GEMSTONE-301 trial, the survival outcomes of consolidation ICI became quite unsatisfactory (13). Of note, in real-world settings, patients with unresectable LA-NSCLC developing large primary tumors (T3-4) or bulky lymph nodes (the shortest diameter ≥ 2cm) are quite common, especially among patients with squamous cell carcinoma (30, 31). Since the CheckMate 816 trial indicated greater survival benefit from neoadjuvant ICI to patients with stage IIIA NSCLC, compared to those with stage IB or II (12), and many previous studies also emphasized the clinical importance of induction or neoadjuvant systematic therapy in patients with bulky diseases, we propose this randomized, phase II trial to investigate whether 2 cycles of induction chemoimmunotherapy could improve survival outcomes in patients with bulky LA-NSCLC (32–34). To the best of our knowledge, the InTRist trial is the first prospective phase II study evaluating induction toripalimab plus chemotherapy in bulky unresectable stage III NSCLC.

In recent decades, the advent of immunotherapy has revolutionized clinical treatment strategies across multiple cancer types. In the context of combined immunotherapy, the remarkable advancements in the therapeutic effect of unresectable stage III NSCLC not only highlight the potential curability of this disease, but shed light on the risk-adaptive multidisciplinary management approach for selective patient population (11, 35). As such, this phase II trial will provide valuable evidence for the personalized comprehensive therapy in LA-NSCLC, and thus may lead to a paradigm shift in LA-NSCLC treatments. By investigating the efficacy and toxicity profile of induction toripalimab combined with chemotherapy in bulky unresectable stage III NSCLC, the InTRist trial is expected to identify and validate a novel and effective therapeutic regimen in this poor-prognostic patient subgroup. Additionally, we will evaluate some promising predictive biomarkers that may be implemented into the clinical practice in the future, and use prospective QoL data to provide practical reference for improving the combination strategy of immunotherapy and radical RT.

Taken together, the InTRist study is the first randomized, phase II clinical trial that probes into the efficacy and safety of 2-cycle induction toripalimab plus chemotherapy followed by curative-intent cCRT and consolidation toripalimab, and compares induction chemoimmunotherapy with chemotherapy alone in the subset of patients with bulky LA-NSCLC. The InTRist trial will offer new insights into the emerging treatment modality of induction anti-PD-1 plus chemotherapy and its clinical usefulness in bulky unresectable stage III NSCLC.

## Ethics statement

All enrolled patients will provide written consent. The ethics committee of the Chinese Academy of Medical Sciences, China, approved the study protocol (No. 22/235-3437). The trial is registered at ClinicalTrials.gov (Identifier: NCT05888402).

## Author contributions

YW: Conceptualization, Data curation, Formal analysis, Investigation, Methodology, Software, Validation, Visualization, Writing – original draft. LD: Conceptualization, Data curation, Formal analysis, Investigation, Methodology, Software, Writing – review & editing. JYW: Conceptualization, Data curation, Formal analysis, Investigation, Methodology, Software, Writing – review & editing. TZ: Conceptualization, Data curation, Formal analysis, Investigation, Methodology, Resources, Validation, Writing – review & editing. WW: Conceptualization, Data curation, Formal analysis, Investigation, Methodology, Resources, Writing – review & editing. XW: Conceptualization, Investigation, Project administration, Resources, Writing – review & editing. WL: Conceptualization, Investigation, Project administration, Resources, Writing – review & editing. YW: Conceptualization, Investigation, Project administration, Resources, Writing – review & editing. JL: Conceptualization, Investigation, Project administration, Resources, Writing – review & editing. QF: Conceptualization, Investigation, Project administration, Resources, Writing – review & editing. ZZ: Conceptualization, Investigation, Project administration, Resources, Writing – review & editing. JW: Conceptualization, Project administration, Supervision, Validation, Writing – review & editing. LW: Conceptualization, Project administration, Supervision, Validation, Writing – review & editing. ZW: Conceptualization, Project administration, Resources, Writing – review & editing. NB: Conceptualization, Funding acquisition, Investigation, Project administration, Writing – review & editing.
